# Assigning value to preparation for prostate cancer decision making: a willingness to pay analysis

**DOI:** 10.1186/s12911-018-0725-4

**Published:** 2019-01-09

**Authors:** Leslie S. Wilson, Traci M. Blonquist, Fangxin Hong, Barbara Halpenny, Seth Wolpin, Peter Chang, Christopher P. Filson, Viraj A. Master, Martin G. Sanda, Gary W. Chien, Randy A. Jones, Tracey L. Krupski, Donna L. Berry

**Affiliations:** 1 University of California San Francisco, 2130 Fulton St, San Francisco, CA 94117 USA; 2 Dana-Farber Cancer Institute, 450 Brookline Avenue, Boston, MA 02215 USA; 3 University of Washington, 1959 NE Pacific St, Seattle, WA 98195 USA; 4 Beth Israel Deaconess Medical Center, 330 Brookline Ave, Boston, MA 02215 USA; 5 Emory University School of Medicine, 1365 Clifton Rd NE, Suite B1400, Atlanta, GA 30322 USA; 6 Kaiser Permanente Medical Center, 4867 Sunset Blvd, Los Angeles, CA 90027 USA; 7 University of Virginia, 202 Jeanette Lancaster Way, Charlottesville, VA 22908 USA

**Keywords:** Prostate cancer, Decision aid, Willingness to pay, Shared decision making

## Abstract

**Background:**

The Personal Patient Profile-Prostate (P3P) is a web-based decision support system for men newly diagnosed with localized prostate cancer that has demonstrated efficacy in reducing decisional conflict. Our objective was to estimate willingness-to-pay (WTP) for men’s decisional preparation activities.

**Methods:**

In a multicenter, randomized trial of P3P, usual care group participants received typical preparation for decision making plus referral to publicly-available, educational websites. Intervention group participants received the same, plus online P3P educational media specific to the user’s personal preferences and values, and a communication coaching component tailored to race\ethnicity, age and language. WTP data were collected one week after physician consultation. An iterative bidding direct contingent valuation survey format was used, randomly assigning participants to high or low starting values (SV). Tobit models were used to explore associations between SV-adjusted WTP and age, education, marital and work-status, insurance, decision-control preference and decision-making stage.

**Results:**

Of 392 participants enrolled, 141 P3P and 107 usual care (UC) provided a WTP value. Men were willing to pay a median $25 (IQR $10–100) for P3P in addition to usual care preparation materials. In the final multivariable tobit regression model, SV, marital status, stage of decision making and income were significantly associated with WTP for P3P. Decision control preference was considered marginally significant (*p* = 0.11). Men were WTP a median $30 (IQR $10–$200) for usual care material alone. In the final multivariable model, SV, education, and stage of decision making were significantly associated with WTP in usual care.

**Conclusion:**

WTP was similar for UC and for the addition of P3P to UC decision preparation. The WTP values were associated with demographic and preference variables. Findings can help focus decision support on future patients who would benefit most: those without strong support systems, at earlier stages of decision making, and open to a shared-decision style.

**Trial registration:**

NCT NCT01844999. Registered May 3, 2013.

**Electronic supplementary material:**

The online version of this article (10.1186/s12911-018-0725-4) contains supplementary material, which is available to authorized users.

## Background

The use of decision aids to promote shared decision making is especially important in prostate cancer treatment decisions, a condition in which personal values and preference regarding risks of treatment side effects influence the patient’s choice [1–3]. The Personal Patient Profile-Prostate (P3P) is a web-based decision support system that has been shown to decrease decisional conflict in men newly diagnosed with localized prostate cancer [4, 5]. Despite evidence of the value of decisions aids, uptake in the United States remains low and economic value may be an important factor. U.S. health care systems, third-party payers and often, providers, want evidence of economic value and patient acceptance before adopting. There is a lack of information about the direct economic value of decision aids precluding providers’ and payers’ consideration of investments in decision aids [6]. There are few economic evaluations of decision aids because the downstream cost savings are often unavailable for measurement. Further, for value-sensitive decisions there are often no right or wrong choices based purely on clinical evidence. However, after engaging with a decision aid, each user can ascertain the value of the aid. Willingness-to-pay (WTP) then becomes an ideal framework for direct economic valuation of decision aids from the user’s viewpoint [7–11] Other non-economic measures, such as decision regret and decisional conflict also can act as measures of an optimal decision choice; [12] these outcomes were part of efficacy testing in our previous clinical trials [4, 5].

There are three contemporary approaches to the measurement of WTP, all of which have been used to find the maximum price a person is willing to pay for a given quantity of a good: 1) contingent valuation, a stated preference approach in which the consumer is directly asked their WTP for a total good; 2) experimental auction, a revealed preference approach where persons are provided actual money and asked to behave in a market of goods, or observed directly in transactions; and 3) conjoint analysis, a stated preference approach where WTP is derived from ranking, rating or selection of goods alternatives by attributes [13]. The choice of method is primarily driven by cost, ease of use, whether or not the good is currently available in the market, the desire for pricing or utility, and whether the estimated WTP is likely to mimic actual market place behavior [14].

The purpose of this study was to estimate clinical trial participants’ WTP for the P3P during the time of decision making for localized prostate cancer care. We also present data on the WTP reported for the control group who received a variety of other ‘usual care’ preparations.

## Methods

### Sample and settings

A multi-center randomized trial was conducted with a primary outcome of decisional conflict. Detailed methods and outcomes were described in a previous publication [5]. Patients were eligible with: 1) a biopsy-proven diagnosis of prostate cancer, cT1 or cT2 of any risk level; 2) an upcoming consult at an enrolling study site; and 3) self-reported ability to read and understand English or Spanish. Participant accrual was from two urology clinics of Kaiser Permanente Southern California, urology and radiation oncology clinics at Beth Israel Deaconess Medical Center in Boston, the University of Massachusetts Memorial Hospital in Worcester, Lyndon B Johnson Hospital urology department in Houston, a community clinic in western New York, four urology clinics in Atlanta’s Emory University network and urology services of University of Virginia Health System. The populations of men with prostate cancer at the sites were diverse regarding race, ethnicity and income [5]. The sites included both membership-based health care institutions, with presumably fewer out of pocket costs, and traditional third-party payer institutions, but out of pocket costs were not measured in this study.

### Design and intervention

While conducting a randomized trial of the P3P Web-based decision aid among men newly diagnosed with localized prostate cancer, we collected WTP data one week after a consult with a prostate cancer physician [15]. The P3P website was accessible on any Web-enabled device for use after the prostate cancer diagnosis and prior to physician consultation. After users answered questions about preferences and decision factors, each was randomized to P3P intervention or usual care (UC). The P3P education and coaching intervention content was targeted to address individuals’ responses, with video clips tailored to race, age and language (English or Spanish). Non-tailored teaching sheets were provided on-screen and in printable format covering eight standard topics in prostate cancer education. Finally, a list of other reputable prostate cancer educational websites was presented to users in both groups. Routinely distributed materials or recommendations in each clinic were provided P3P group participants were asked their WTP for only the P3P intervention, while the UC group was asked their WTP for the list of websites and any other education they reported receiving or finding We considered WTP for P3P to be additional to this education at each site as all participants had access to these resources.

### WTP approach: Stated preference vs revealed preference

The direct contingent evaluation WTP approach was selected for this study, first, for its ease of use when eliciting information from patients at a stressful time after a cancer diagnosis. Secondly, P3P was not on the market for evaluation by actual use and was not directly paid for by the patient, precluding measuring market behavior. Finally, because the decision aid offered education in a context where there is no right or wrong choice, the use of the other methods to assess optimal choice or medical savings was not possible. In addition, we were interested in a direct price value, rather than price as one factor of a mixed utility evaluation (e.g., conjoint analyses). Despite some methodological limitations of WTP approach to measure more theoretical environmental value questions, it remains a favored approach for determining health care value [16, 17].

### Telephone survey

An iterative bidding direct contingent valuation survey format, delivered by telephone one week after consultation with a physician, was used to elicit the WTP. Participants were randomly assigned, stratified by institutional site, to either a high ($200) or low ($10) starting value (SV) to enable control for starting point bias. The starting points were based on a previous WTP study of a decision aid used in breast cancer patients [18]. Based on the first WTP value, participants were asked if they were willing to pay alternately higher and lower values until the maximum WTP was reached, at which point the final maximum WTP was confirmed. While bidding game formats may result in higher valuations than open ended payment formats, all suffer from anchoring effects. Bidding formats exclusively obtain the maximum WTP, whereas payment ranges eliminate knowing if a person’s choice is a maximum or minimum payment preference [19].

The survey was delivered by telephone to both the P3P intervention and usual care (UC) groups. A research coordinator read scripted instructions and asked respondents to consider the materials they were being asked to value. The P3P group participants were reminded of using the intervention and then were asked “Would you be willing to pay $[200/10] to use P3P?” In the UC group, the script included instructions to describe any materials provided by their hospital, clinic or providers, then reminded them of the standard educational websites they have been given in the P3P program; respondents were then asked “Would you be willing to pay [$200/$10] for the materials from your hospital/clinic and the websites from P3P?”

### Analysis

Fisher’s exact test was used to evaluate differences in patient characteristics between participants who did and did not provide a WTP value. Reasons for not providing a WTP value were investigated and described qualitatively. The team decided a priori to analyze WTP separately for the P3P and UC groups to determine the value which participants were willing to pay under the different circumstances of educational support. Associations between SV and the continuous WTP value were evaluated with the Wilcoxon rank sum test. Based on recommendations to match the measure with the purpose of its use, [20] median WTP values were summarized. A WTP demand curve was created by plotting the number of participants willing to pay above a given dollar amount.

Due to the right-skew in WTP values, a log (WTP + 1) transformation was performed and outliers were removed. Tobit regression models, left censored at a value of $0, were used to explore the association between SV-adjusted WTP and: age (< 60 years, 60–69 years, ≥70 years), education (≤high school, >high school), marital status (not married/partnered, married/partnered), work status (working, not working), income (<$40,000, $40,000–$99,999, ≥$100,000), insurance (Medicare, private, other), decision control preference (active/shared, or passive decision making), and decision making stage (not started, started, close to deciding/decided). Multivariable model-building followed a forward stepwise selection method in which SV was retained. The selection criteria utilized *p*-values with significance levels of 0.2 to be entered and 0.1 to be retained in the model. Tobit regression models were fit using the *VGAM* [21] package in R version 3.4.3 [22]. All other analyses were performed in SAS software version 9.4 of the SAS System for Windows, PC, © 2013, SAS Institute Inc [23].

## Results

Of 577 men contacted, 415 (72%) enrolled and 392 eligible participants were randomized in the clinical trial from September 2013 through April 2016. [5] We attempted to reach all participants by phone 1-week after the physician consult for the WTP survey. Sixty-six participants (17%) were not reached; 18 (5%) refused to complete the survey; and 60 (15%) agreed to the survey but were unable or unwilling to provide a WTP value (Table 1). The total number of participants providing a WTP value was 248 (63%). Overall, not providing a WTP value was associated with being randomized to UC (*p* = 0.001) and, regardless of study group, having high school or lower education (*p* = 0.047).

**Table 1:** Participant characteristics

	Overall	Provide WTP Amount^b^				Study Group^c^			
		No		Yes		P3P		UC	
	N	N	%	N	%	N	%	N	%
N	392	144	37	248	63	141		107	
Study Group									
Control	194	87	45	107	55				
Intervention	198	57	29	141	71				
Age									
< 59 years	125	42	34	83	66	48	34	35	33
60–69 years	196	77	39	119	61	65	46	54	50
≥ 70 years	71	25	35	46	65	28	20	18	17
Education (*n* = 387)									
≤ High school	77	36	47	41	53	20	14	21	20
> High school	310	105	34	205	66	120	85	85	79
Marital Status (*n* = 389)									
Not married/Partnered	109	43	39	66	61	40	28	26	24
Married/Partnered	280	99	35	181	65	101	72	80	75
Work Status (*n* = 387)									
Not working	162	58	36	104	64	51	36	53	50
Working	225	83	37	142	63	88	62	54	50
Income (*n* = 355)									
< $40,000	97	37	38	60	62	35	25	25	23
$40,000–$99,999	120	46	38	74	62	39	28	35	33
≥ $100,000	138	42	30	96	70	57	40	39	36
Insurance (*n* = 387)									
Medicare	116	37	32	79	68	43	30	36	34
Private	194	68	35	126	65	76	54	50	47
Other^a^	77	36	47	41	53	22	16	19	18
Preference (*n* = 381)									
Active	126	39	31	87	69	50	35	37	35
Passive/Shared	255	97	38	158	62	89	63	69	64
Stage of Decision (*n* = 387)									
Not started	87	31	36	56	64	36	26	20	19
Started	187	66	35	121	65	64	45	57	53
Close/decided	113	45	40	68	60	39	28	29	27

WTP = willingness to pay

^a^Other includes: none, public/private, military, Medicaid

^b^Row percentages ^c^Column percentages

### WTP for P3P intervention

In the intervention group (*n* = 198), we successfully contacted and obtained a WTP value on the 1-week WTP survey for 141 (71%) participants. Among the 57 not providing a value, 32 (56%) were not reached, 6 (10%) refused the questionnaire, and 19 (33%) agreed to answer the questionnaire but did not provide a final WTP value. Of these 19 men, 12 reported not spending any time using P3P, two who spent less than an hour using P3P, one who reported using P3P for 1 to 2 h but could not determine a value, and one who reported more than 4 h use and that the information in P3P was “worth a lot,” but he could not assign a monetary value. Three men did not report time spent using P3P or the reason(s) for not assigning a value. Participant characteristics for those providing a WTP value in the P3P intervention group are presented in Table 1. No significant differences were detected between those who did or did not provide a WTP value (data not shown). Amount of time spent using P3P was known for 133 participants; the majority (59%) spent less than 1 h using P3P, while 44 (33%) spent 1 to 2 h.

**Fig. 1:**
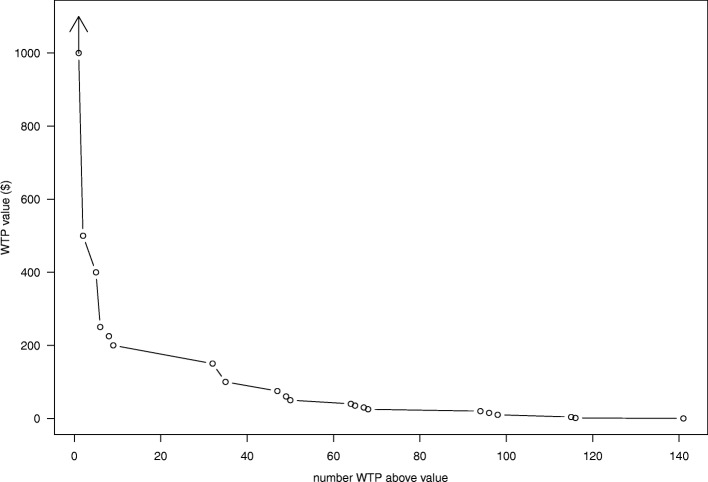
WTP demand curve for the P3P intervention

Of the 141 participants who gave a WTP value, 24 (17%) reported $0. Participants who chose to comment on the $0 value indicated *unwillingness* to pay for information available on the Internet (*n* = 5) or that sufficient information was gathered prior to using P3P (n = 5). The remainder reported a WTP from $1.29 to $100,000. The participant reporting a $100,000 value stated P3P was “beyond helpful;” this value was 100 times larger than the next highest value reported. Additionally, the value was more than two standard deviations from the transformed mean and was therefore treated as an outlier in all tobit models. For all reporting, the median WTP for P3P was $25 (IQR $10–100) (Additional file 1: Table S1). Overall, WTP value was associated with SV (*p* = 0.018); the median WTP value in those with a low and high SV was $25 (IQR $10–$75) and $50 (IQR $22.5–$200), respectively. The WTP demand curve (Fig. 1) has a steep slope at the highest WTP values and then a flatter curve across other WTP values.

In SV-adjusted univariate tobit regression models, marital status, decision control preference, stage of decision, and income were significantly associated with the predicted WTP value (Additional file 2: Table S2). In the final multivariable model, starting value, marital status, stage of decision making, and income retained significance with *p*-values < 0.10, and decision preference was considered marginally significant (*p* = 0.11). The resulting model indicated the predicted WTP values for participants randomized to a high SV (compared to a low SV) and not married/partnered (compared to married/partnered) were 2.15 (90% CI 1.22–3.78) and 2.77 (90% CI 1.26–6.05) times higher, respectively. The predicted WTP value for participants who had not yet started, or had just started the decision-making process, was 2.81 (90% CI 1.34–5.89) and 2.34 (90% CI 2.34–4.53) times higher, respectively, than participants who either decided or were close to a decision at study entry. The predicted WTP value for participants with an income $40,000–$99,999 was 2.39 (90% CI 1.23–4.66) times higher than those with an income ≥$100,000; no other significant differences with income were detected (Table S2). The predicted WTP value for participants with a shared/passive decision-making preference was marginally higher, 1.75 (90% CI 0.98–3.11) times compared to those with an active decision-making preference.

### WTP for usual care

In UC (*n* = 194), we reached 107 (55%) men who also reported a WTP value for decision preparation materials. Of the 87 who did not provide a value, 34 (39%) were not reached, 12 (14%) refused to answer the questionnaire, and 41 (47%) agreed to answer the questionnaire but did not provide a final WTP value. Among the last group, the most common reason for not providing a WTP value was that no materials were reviewed. Of the 107 participants who provided a value, 99 indicated the type of education support they received, of which the most commonly used were websites (*n* = 81) and brochures/pamphlets (*n* = 50). The majority (67%) spent less than 2 h using these materials. No significant differences were detected between those who provided and did not provide a final WTP value. The median WTP for UC was $30 (IQR $10–$200) with a range of $0 to $5000 (Table 1). WTP was associated with SV (*p* = 0.031), with a median WTP of $25 (IQR $0–$75) and $50 (IQR $22–$200) in low and high SV respectively.

In the SV-adjusted, univariate tobit regression models, education and stage of decision making were significantly associated with the WTP (Additional file 3: Table S3). The final multivariable tobit model included starting value, education, and stage of decision making. The predicted WTP values for those randomized to a high starting value (compared to a low SV) and with a < high school education (compared to > high school education) were 2.35 (90% CI 1.10–5.01) and 3.59 (90% CI 1.36–9.45) times higher, respectively. The predicted WTP value was 0.30 (0.12–0.70) times lower for those who had just started the decision-making process compared to those who were close to a decision/already decided at study entry. A sensitivity analysis was conducted removing $5000, a value more than two standard deviations from the transformed mean; results remained consistent (Additional file 3: Table S3).

## Discussion

The majority of participants in both study groups readily reported WTP for decision preparation, for the P3P intervention and for the various materials provided to participants in the UC group. Participants in both study groups were willing to pay a median value ranging from $25–50, depending on SV. Demographic variables and participants’ baseline stages of decision making predicted various WTP values suggesting differential benefit for individual characteristics.

Participant characteristics related to higher WTP values suggest which patients likely need greater decisional support. Those not married or partnered valued P3P more; men with fewer personal resources may value a decision aid more highly than a man with a partner who can share in the decision-making process. Those who had not yet or just started decision making logically indicated a higher value for decision support. Finally, the finding that participants are willing to pay more for P3P if they prefer to make shared rather than passive decisions is important. This valuation could mean that participants who were not yet ready to engage with a decision aid, had a more private decision-making style or found less value in decision aids. These WTP valuations of P3P can help to focus on future patients who would benefit most from decision aids: men not yet starting decision making, those without a partner/spouse to share in the decision process, and those open to shared decision making. Increased WTP for P3P in both lower and higher incomes, versus moderate incomes, differs from the expected direct association of WTP with ability to pay. Further work could explore detailed financial resources and WTP responses.

The demand curve for WTP demonstrated that the approach performed well in economic terms. The curve flattened at lower prices as the demand became more elastic in the presence of low-cost education substitutes.

In the UC group, the type and extent of preparatory educational materials reported was variable. It was likely more difficult to provide a consistent WTP value for a collection of various educational materials. Our finding that a lower proportion of UC participants, as compared to P3P users, were able to provide a WTP value supports this implication.

The participant characteristics associated with WTP for usual care differed from those for P3P. For UC, those with a lower level of education expressed higher WTP; perhaps perceiving a greater need for decision materials provided (or not) by the consulting physician’s office. In contrast to the P3P group, UC respondents who had already decided were willing to pay more than those who had not yet or just started their decision making. This counter-intuitive result might indicate that participants who made a decision even before meeting the physician were willing to pay more for a diverse collection of non-personalized material, while those unable to make a quick decision with the available material valued such materials less. Patients will always have access to a variety of ad hoc and Internet sources and may have difficulty determining quality.

Limitations in our study are those inherent in many WTP studies: protest responses (nonresponses when unwilling to provide a value or for some zero values), preference uncertainty or the confidence of one’s WTP response, and outliers [7, 24–29]. Non-response, infinity (very high valuations) and zero responses are not uncommon in WTP assessments [7, 27–29] and are difficult to interpret. Non-response is often from a lack of exposure to the product, as was the case in our study, but it also can represent a protest response, an expression of preference that is not reflective of economic assumptions underlying WTP theory [27, 28]. Halstead, et al. and others [27, 30] found that censoring protest responses should only occur if the characteristics of the protest responders do not differ significantly from the other respondents. Our comparisons of responders and non-responders found no particular characteristic for bias.

Zero responses also can be either a true zero WTP value or a protest vote. For example, some of the P3P participants’ reasons for giving a zero value, unwillingness to pay for available internet information, suggested a protest WTP response. Although some methods have been described modeling zero values, or protest responses, to be conservative we included all zero WTP responses in our estimates [7, 27, 31]. Preference uncertainty may be caused by incomplete knowledge about product features or just uncertainty about their own preferences [32]. WTP values are less valid when preference uncertainty is high, such as for the UC’s undefined materials, as evidenced in part by the 44% not providing a WTP value.

Another limitation, common to WTP studies is the presence of outliers. We had one main outlier in the P3P group who expressed a WTP value 100 times greater than the next highest value. There is no consensus on how outliers should be handled in WTP studies; the method largely depends on how the WTP results will be used. For analysis of characteristics associated with WTP values, removing the outliers provides the most accurate results, as we did here in all our means-based analyses. However, for inclusion in cost-benefit analyses, it is suggested to retain those outliers to represent the full range of the benefit of the intervention. An alpha-trimmed mean can also be considered [33].

Our results are limited by estimating the value of P3P alone as an adjunct to usual patient education. Although P3P’s value may be additive to usual education, we cannot assume that the value of usual education would remain constant when P3P is also used.

A strength of our study was that we measured and controlled for starting point bias, common but not universal in WTP surveys that use a bidding approach. Similar to our finding that the P3P group starting with a high value was willing to pay about $42 more than those starting low, Stalhammar [34] found a starting point bias, with a WTP of only $9 vs $38 for a pill taken at meals instead of before meals depending on starting with a low or high amount, respectively. Another study evaluating WTP for treatment of bleeding disorders found a median WTP of only $1500 when bidding started at $500 and $3500 if started at $5000 [8]. Starting point bias is not fully eliminated by any method that provides a direct WTP value. Future studies can use multiple approaches to explore the effects of different WTP questionnaire methods [19]. Although conjoint analyses are becoming more popular for measuring patient preferences in health care, their WTP responses are not independent of other measure attributes included in a conjoint approach.

There are very few economic studies evaluating decision aids, and fewer that use WTP for evaluating cost-benefit. One such study used WTP to determine the cost-benefit of the use of a decision support program for patients with breast cancer in a rural community [18]. This program included a trained facilitator who met with the participant before a physician visit and provided a personalized written “consultation plan” to use during the visit. WTP averaged $150, however the cost of this labor-intensive program was high, resulting in a positive net-benefit only when the most efficient parameters were considered. We anticipate that the P3P program will have a more efficient implementation cost due to its intrinsic automation and be able to demonstrate a positive net-benefit given the high mean WTP value from participants. As WTP for P3P reported here was a demonstration of the perceived value, in future analyses we will combine this with the costs for P3P program delivery across delivery sites to determine the overall value of the P3P program in a net benefit calculation.

The WTP expressed by men making decisions after a diagnosis of localized prostate cancer demonstrated a valued decision aid, tailored to users’ personal preferences and concerns and always available for return visits. Such availability is important, given the frequent reports that patients are often not prepared for cancer consultations [18, 35], or do not remember the content of these meetings [10, 36, 37]. The WTP for P3P was instructive, especially when further characterized by influence of demographic characteristics. Our findings suggest that single men or those with modest incomes are likely targets for decisional support. Further, men who are open to a shared decision-making style are likely eager recipients of decision support. Health care systems with limited resources could target groups with the greatest need for decision preparation and provide P3P.

## Conclusions

Our study used the WTP contingent valuation technique to demonstrate that men with localized prostate cancer who were randomized to P3P, a personalized web-based decision support system, or to UC, were readily willing to pay for a decision aid. We demonstrated P3P WTP valuations that can help providers and health care systems focus on future patients who would most value P3P: men who have not yet started to make a decision, those without a partner or spouse to share the decision process, and those open to shared decision making. These results comprise the evidence needed by payers to support the adoption of P3P to support men making decisions about prostate cancer treatments.

## Additional files

Additional file 1: **Table S1.** Actual, Transformed and Back-Transformed Willingness to Pay values for P3P and Usual Care Groups by High or Low Starting Values. Summary of the WTP values for each study group; new table requested by reviewers. (DOCX 13 kb)

Additional file 2: **Table S2.** Tobit Regression Model for P3P Intervention- All Values <$100,000. Table detailing the starting value adjusted univariate and final multivariable for the UC group. (DOCX 17 kb)

Additional file 3: **Table S3.** Tobit Regression Model for Usual Care. Table detailing the starting value adjusted univariate and final multivariable for the P3P group. (DOCX 17 kb)

## Abbreviations

P3P: Personal Patient Profile-Prostate
SV: starting values
UC: usual care
WTP: willingness-to-pay
