# Supplementary material for: Assigning value to preparation for prostate cancer decision making: a willingness to pay analysis
Source: BMC Med Inform Decis Mak. 2019 Jan 9;19:6. doi: 10.1186/s12911-018-0725-4 (PMC6327504; doi:10.1186/s12911-018-0725-4)
Supplement: Supplementary file 1 — Table S1. Actual, Transformed and Back-Transformed Willingness to Pay values for P3P and Usual Care Groups by High or Low Starting Values. Summary of the WTP values for each study group; new table requested by reviewers. (DOCX 13 kb) [file 12911_2018_725_MOESM1_ESM.docx]

**Supplemental Table 1:** Actual, Transformed and Back-Transformed Willingness to Pay values for P3P and Usual Care Groups by High or Low Starting Values

|  | No.  reporting | Actual WTP Values | | Transformed | Back-transformed |
| --- | --- | --- | --- | --- | --- |
|  |  | Range | Median (IQR) | Mean (SD) | Mean (95% CI) |
| P3P |  |  |  |  |  |
| All WTP values | 141 | $0-100,000 | $25 ($10-100) | 3.36 (1.97) | $27.76 ($19.72-$38.92) |
| Start High | 72 | $0-100,000 | $50 ($22.5-$200) | 3.74 (2.03) | $41.00 ($25.04-$66.76) |
| Start Low | 69 | $0-$500 | $25 ($10-$75) | 2.96 (1.83) | $18.37 ($11.48-$29.06) |
| WTP <$100,000 | 140 | $0-1,000 | $25 ($10-100) | 3.30 (1.85) | $26.13 ($18.92-$35.96) |
| Start High | 71 | $0-$1,000 | $50 ($20-200) | 3.63 (1.82) | $36.65 ($23.45-$56.97) |
| Start Low | 69 | $0-$500 | $25 ($10-$75) | 2.96 (1.83) | $18.37 ($11.48-$29.06) |
| UC |  |  |  |  |  |
| All WTP values | 107 | $0-$5,000 | $30 ($10-200) | 3.38 (2.01) | $28.46 ($19.04-$42.31) |
| Start High | 56 | $0-$1,000 | $50 ($22.5-$200) | 3.76 (1.87) | $41.97 ($25.06-$69.87) |
| Start Low | 51 | $0-$5,000 | $25 ($0-$75) | 2.97 (2.10) | $18.46 ($9.79-$34.10) |
| WTP <$5,000 | 106 | $0-$1,000 | $27.5 ($10-200) | 3.33 (1.96) | $27.07 ($18.26-$39.91) |
| Start High | 56 | $0-$1,000 | $50 ($22.5-$200) | 3.76 (1.87) | $41.97 ($25.06-$69.87) |
| Start Low | 50 | $0-$500 | $25 ($0-$75) | 2.86 (1.96) | $16.42 ($8.98-$29.41) |

WTP=willingness to pay; IQR=Interquartile range; SD=standard deviation; CI=confidence interval; P3P=Personal Patient Profile-Cancer; UC=Usual care
