# Supplementary material for: Assigning value to preparation for prostate cancer decision making: a willingness to pay analysis
Source: BMC Med Inform Decis Mak. 2019 Jan 9;19:6. doi: 10.1186/s12911-018-0725-4 (PMC6327504; doi:10.1186/s12911-018-0725-4)
Supplement: Supplementary file 2 — Table S2. Tobit Regression Model for P3P Intervention- All Values <$100,000. Table detailing the starting value adjusted univariate and final multivariable for the UC group. (DOCX 17 kb) [file 12911_2018_725_MOESM2_ESM.docx]

**Supplemental Table 2:** Tobit Regression Model for P3P Intervention- All Values <$100,000

|  |  | SV-Adjusted  Univariate Models | | Final Multivariable  Model | | |
| --- | --- | --- | --- | --- | --- | --- |
| Covariate | Levels | L. Est. | p | L. Est. | p | Exp(L. Est.)  [90% CI] |
| Starting Value (SV) | High vs.  low | 0.74 | 0.042 | 0.77 | 0.025 | 2.15  [1.22-3.78] |
| Marital status | No vs.  yes | 1.02 | 0.009 | 1.02 | 0.032 | 2.77  [1.26-6.05] |
| Decision  preference | Shared/passive vs.  active | 0.74 | 0.049 | 0.56 | 0.11 | 1.75  [0.98-3.11] |
| Stage of  decision | Not started vs.  made decision | 1.17 | 0.015 | 1.03 | 0.022 | 2.81  [1.34-5.89] |
|  | Started vs.  made decision | 0.89 | 0.037 | 0.85 | 0.033 | 2.34  [1.21-4.53] |
| Income | <$40K vs.  ≥$100K | 1.20 | 0.005 | 0.37 | 0.48 | 1.44  [0.61-3.40] |
|  | $40K-$100K vs.  ≥$100K | 1.09 | 0.009 | 0.87 | 0.032 | 2.39  [1.23-4.66] |
| Insurance | Private vs.  Medicare | -0.82 | 0.044 | - | - | - |
|  | Other vs.  Medicare | -1.31 | 0.019 | - | - | - |
| Work status | Yes vs.  no | -0.87 | 0.016 | - | - | - |
| Age | <59 years vs.  ≥70 years | -0.78 | 0.13 | - | - | - |
|  | 60-69 years vs.  ≥70 years | -0.59 | 0.22 | - | - | - |
| Education | >High school vs.  ≤High school | 0.32 | 0.54 | - | - | - |

L. Est. = Linear Estimate

Exp(L. Est.) = exponentiated (back transformed) linear estimate
