# Supplementary material for: Assigning value to preparation for prostate cancer decision making: a willingness to pay analysis
Source: BMC Med Inform Decis Mak. 2019 Jan 9;19:6. doi: 10.1186/s12911-018-0725-4 (PMC6327504; doi:10.1186/s12911-018-0725-4)
Supplement: Supplementary file 3 — Table S3. Tobit Regression Model for Usual Care. Table detailing the starting value adjusted univariate and final multivariable for the P3P group. (DOCX 17 kb) [file 12911_2018_725_MOESM3_ESM.docx]

**Supplemental Table 3:** Tobit Regression Model for Usual Care

|  |  | SV-Adjusted  Univariate Models | | Final Multivariable  Model | | | Final Multivariable Model  (<$5,000 WTP values) | | |
| --- | --- | --- | --- | --- | --- | --- | --- | --- | --- |
| Covariate | Levels | L. Est. | p | L. Est. | p | Exp(L. Est.)  [90% CI] | L. Est. | p | Exp(L. Est.)  [90% CI] |
| Starting Value (SV) | High vs.  low | 0.96 | 0.036 | 0.85 | 0.063 | 2.35  [1.10-5.01] | 0.95 | 0.033 | 2.56  [1.24-5.30] |
| Education | ≤High school vs.  >High school | 1.11 | 0.053 | 1.28 | 0.030 | 3.59  [1.36-9.45] | 1.39 | 0.014 | 4.01  [1.58-10.16] |
| Stage of  decision | Not started vs.  made decision | -0.66 | 0.33 | -0.93 | 0.18 | 0.39  [0.13-1.23] | -1.30 | 0.054 | 0.27  [0.09-0.83] |
|  | Started vs.  made decision | -1.11 | 0.037 | -1.22 | 0.021 | 0.30  [0.12-0.70] | -1.22 | 0.015 | 0.29  [0.13-0.67] |
| Marital status | Yes vs.  no | 0.27 | 0.62 | - | - | - | - | - | - |
| Decision  preference | Shared/passive vs.  active | 0.37 | 0.45 | - | - | - | - | - | - |
| Income | <$40K vs.  ≥$100K | 0.26 | 0.68 | - | - | - | - | - | - |
|  | $40K-$100K vs.  ≥$100K | 0.38 | 0.50 | - | - | - | - | - | - |
| Insurance | Medicare vs.  Private | -0.38 | 0.46 | - | - | - | - | - | - |
|  | Other vs.  Private | -1.13 | 0.083 | - | - | - | - | - | - |
| Work status | Yes vs.  no | -0.58 | 0.20 | - | - | - | - | - | - |
| Age | <59 years vs.  ≥70 years | 0.43 | 0.53 | - | - | - | - | - | - |
|  | 60-69 years vs  ≥70 years | 0.46 | 0.48 | - | - | - | - | - | - |

L. Est. = Linear Estimate

Exp(L. Est.) = exponentiated (back transformed) linear estimate
